# Supplementary material for: Analysis of PPARγ Signaling Activity in Psoriasis
Source: Int J Mol Sci. 2021 Aug 10;22(16):8603. doi: 10.3390/ijms22168603 (PMC8395241; doi:10.3390/ijms22168603)
Supplement: Supplementary file 1 [file ijms-22-08603-s001.zip › Supplemental materials_Analysis of PPARg signaling activity in psoriasis/Pathway models/Models images and html files/PPARG expressed in cells assotiated with psoriasis/225587.html]

melanocyte


# CellType melanocyte

|  |  |
| --- | --- |
| URN | urn:agi-ncimcelltype:C0025201 |
| Total Entities | 2 |
| Connectivity | 2404 |
| Name | melanocyte |

---

|  |  |
| --- | --- |
| ChildConcepts | melanoblast |
|  | nevus cell |

---

|  |  |
| --- | --- |
| Pathway | EGFR -> Expression Targets in Skin |
|  | ITGB1 -> Expression Targets in Skin |
|  | NOTCH -> Expression Targets in Skin |
|  | PPARG expressed in cells assotiated with psoriasis |

---

|  |  |
| --- | --- |
| MedScan ID | 10000106 |

---

|  |  |
| --- | --- |
| Alias | epidermis melanocyte |
|  | melanotic cells |
|  | melanocyte |
|  | melanocytes |
|  | epidermis melanocytes |
|  | melanocytic |
|  | melanotic cell |

---

|  |  |
| --- | --- |
| NCIm ID | C0025201 |

---
